# Once upon a time, there was a fabulous funambulist…: what children learn about the “high-level” vocabulary they encounter while listening to stories

**DOI:** 10.3389/fpsyg.2014.00075

**Published:** 2014-02-07

**Authors:** Carmel Houston-Price, Jodie A. Howe, Natalie J. Lintern

**Affiliations:** School of Psychology and Clinical Language Sciences, University of ReadingReading, UK

**Keywords:** vocabulary, comprehension, listening to stories, definitions, grammaticality judgments, teacher-led intervention, word learning, forced-choice

## Abstract

Previous research has shown that listening to stories supports vocabulary growth in preschool and school-aged children and that lexical entries for even very difficult or rare words can be established if these are defined when they are first introduced. However, little is known about the nature of the lexical representations children form for the words they encounter while listening to stories, or whether these are sufficiently robust to support the child's own use of such “high-level” vocabulary. This study explored these questions by administering multiple assessments of children's knowledge about a set of newly-acquired vocabulary. Four- and six-year-old children were introduced to nine difficult new words (including nouns, verbs and adjectives) through three exposures to a story read by their class teacher. The story included a definition of each new word at its first encounter. Learning of the target vocabulary was assessed by means of two tests of semantic understanding—a forced choice picture-selection task and a definition production task—and a grammaticality judgment task, which asked children to choose between a syntactically-appropriate and syntactically-inappropriate usage of the word. Children in both age groups selected the correct pictorial representation and provided an appropriate definition for the target words in all three word classes significantly more often than they did for a matched set of non-exposed control words. However, only the older group was able to identify the syntactically-appropriate sentence frames in the grammaticality judgment task. Further analyses elucidate some of the components of the lexical representations children lay down when they hear difficult new vocabulary in stories and how different tests of word knowledge might overlap in their assessment of these components.

## Introduction

Having a large and varied vocabulary at one's disposal confers a significant advantage in adulthood, and the same is true for preschool- and young school-aged children. The size of a young child's vocabulary predicts later language competence, reading comprehension, writing skills and general academic achievement (Cunningham and Stanovich, 1997; Storch and Whitehurst, 2002; Dickinson et al., 2003). As described by Chall and Jacobs (2003), the successful development of general knowledge, literacy, and other academic skills depends fundamentally on the child having a sufficient grasp of the language in which they are attempting to learn. Moreover, the benefits of possessing a large vocabulary continue to accrue if the child progresses beyond the level required to meet age-appropriate targets; it is the children with the most sophisticated language skills who show the greatest academic achievements across the curriculum. It has therefore been argued that research should focus on identifying how growth in children's knowledge of “high-level” (or “Tier 2”) vocabulary (i.e., words beyond the expected level for the child's chronological age) is best supported (Beck et al., 2002).

In some cases, children acquire rich and diverse vocabulary knowledge through the conversational exchanges that surround everyday activities at home. Beals (1997) analyzed the content of meal-time conversations between parents and their 3- to 5-year-old children and found that the frequency with which parents used rare words and the extent to which they provided “semantic support” for these words (e.g., by defining the word's meaning) predicted the child's performance on standardized tests of vocabulary knowledge at 5 and 7 years of age. Weizman and Snow (2001) similarly found that parents' use of sophisticated vocabulary in conversations with their 5-year-old children—again measured in terms of the number of high-level words used by parents and the level of support they provided children in interpreting these words—explained 40% of the variance in children's vocabulary scores at second grade, after parental education, child language and child non-verbal IQ scores had been controlled for. These studies demonstrate the important role parents play in helping their child to build a large vocabulary. However, many parents will be unable to provide the sophisticated linguistic environment necessary to support the development of high-level vocabulary; the children in these families are therefore dependent on alternative sources of lexical variety.

Listening to stories has long been acknowledged as an activity that supports vocabulary acquisition in young children (Ninio, 1983; Elley, 1989). Numerous studies have demonstrated gains in word knowledge to result from storytelling to preschoolers (Senechal and Cornell, 1993; Sénéchal et al., 1995; Karweit and Wasik, 1996; Reese and Cox, 1999; Wasik and Bond, 2001; Justice et al., 2005; Walsh and Blewitt, 2006; Wasik et al., 2006) and school-age populations (Dickinson, 1984; Nagy et al., 1987; Elley, 1989; Robbins and Ehri, 1994; Penno et al., 2002; Wilkinson and Houston-Price, 2013). Entry to formal education provides children with further opportunities to engage in activities that support the acquisition of vocabulary knowledge, including explicit instruction and classroom discussion about specific vocabulary items (Beck et al., 1982; Stahl and Fairbanks, 1986; Beck et al., 2002) and, with the onset of literacy, the chance to encounter new words in written texts (Jenkins et al., 1984; McKeown et al., 1985; Nagy et al., 1987). However, children continue to learn new words by listening to spoken language, and to stories in particular, throughout the school years (Dickinson, 1984; Nagy et al., 1987; Elley, 1989; Robbins and Ehri, 1994; Penno et al., 2002; Wilkinson and Houston-Price, 2013); listening to stories is “almost universally praised” (Elley, 1989, p. 176) as a means of promoting vocabulary growth. There are, of course, individual differences in children's ability to profit from listening to stories; many studies have demonstrated the “Matthew effects” (Stanovich, 1986) that are ubiquitous in children's development (Robbins and Ehri, 1994; Sénéchal et al., 1995; Reese and Cox, 1999). However, recent work has shown that children of all ages and abilities are able to learn difficult new words from age-appropriate stories (Wilkinson and Houston-Price, 2013), suggesting that classroom story sessions are a truly “democratic” learning activity.

A key determinant of the success of children's learning of new vocabulary while listening to stories is the manner in which new words are presented. While some degree of learning can result from a single implicit exposure to a new word in context (Dickinson, 1984; Stahl et al., 1991), many studies have shown that learning is facilitated by repeated exposure, whether through repetition of the vocabulary within a story or through repeated readings of the same story (Elley, 1989; Robbins and Ehri, 1994; Karweit and Wasik, 1996; Sénéchal, 1997; Beck and McKeown, 2007). Learning is also supported when the reader provides opportunities for the child to engage interactively with the new vocabulary that they meet. Posing questions that include the new word or that require the child to produce the new word as an answer and engaging in role-play involving the new word all serve to facilitate learning (Sénéchal et al., 1995; Sénéchal, 1997; Ewers and Brownson, 1999; Ard and Beverly, 2004; Walsh and Blewitt, 2006; Blewitt et al., 2009). And just as children benefit from explicit clarification of the meanings of the new vocabulary items they encounter in conversation (Beals, 1997; Weizman and Snow, 2001), they also show greater learning of the vocabulary they meet when listening to stories if the reader provides an age-appropriate definition or synonym for the word, or points to an enlightening illustration to explain its meaning, while reading the text (Elley, 1989; Sénéchal et al., 1995; Brett et al., 1996; Sénéchal, 1997; Reese and Cox, 1999; Penno et al., 2002; Justice et al., 2005; Biemiller and Boote, 2006; Beck and McKeown, 2007; Wilkinson and Houston-Price, 2013).

Recent research from our group has shown that listening to stories including explicit definitions supports children in the acquisition of very difficult vocabulary. Wilkinson and Houston-Price (2013) asked teachers to read stories containing a set of eight high-level words to their Year 2 and 4 classes (7- and 9-year-olds) once a week for 3 weeks. In some conditions, the first mention of each word was accompanied by a child-friendly definition of its meaning. Children were tested on their understanding of the target vocabulary and a matched set of control words three times: at baseline, immediately after the 3-week exposure phase, and again 2 weeks later, to establish the extent to which children retained their learning. Substantial gains in understanding of the target words were made during the listening phase, and these were maintained at least 2 weeks after the story was last heard. Interestingly, analyses of children's longer-term learning showed that the two age groups made equivalent gains in vocabulary knowledge although, as in previous demonstrations of the Matthew Effect (Stanovich, 1986), the greatest gains were shown by children with more advanced vocabulary knowledge, according to their scores on the British Picture Vocabulary Scale (BPVS-II; Dunn et al., 1997). The provision of definitions during the story reading sessions boosted children's understanding of the new words regardless of their age or prior vocabulary knowledge, confirming the importance of providing “semantic support” for high-level vocabulary when it is first introduced.

In Wilkinson and Houston-Price's (2013) study, learning was assessed in terms of children's ability to select the correct pictorial representation of the new words from an array of four pictures; we borrowed both the target vocabulary and the forced-choice test cards for these words from the BPVS-II for this purpose. The benefit of this type of assessment is that one can compare the number of correct selections children make for target words to the number expected by chance, or to performance on a set of control words matched for difficulty. However, this type of measure can provide only limited information about the nature of the learning that has taken place. Correct performance on a forced-choice task requires only a gist understanding of a word's meaning; a child might succeed on such a task on the basis of a rather sketchy representation of a word's semantics, or even a vague notion of the contexts in which a word is *not* appropriate. A word might more reasonably be said to be “known” if it is stored in the lexicon in such a way as to allow the child to use it in their expressive and receptive language.

Other work in this field has assessed learning by asking participants to provide a synonym or definition of the target vocabulary (e.g., Dickinson, 1984; Steele et al., 2012). The requirement to produce an appropriate definition provides a more conservative test of word knowledge than picture selection, as it challenges the child to retrieve the semantic information stored alongside the word's lexical entry. Success at such a task would not be supported by a partial representation of a word's meaning, as it could in a forced-choice task. However, the ability to produce a suitable definition or synonym also depends on expressive language competence; the full extent of the child's semantic representation of a word might therefore not be evident from their response. As no single measure can provide a comprehensive picture of the lexical representation a child holds for a word, Dockrell et al. (2007) propose that children's learning should be investigated using multiple assessments of word knowledge.

One approach to providing a comprehensive picture of children's learning is to probe their awareness of the newly-acquired word's semantic properties through a graduated or “dynamic” assessment method (Gutiérrez-Clellen and Peña, 2001). For example, to test children's understanding of vocabulary encountered while reading stories, Steele et al. (2012) first asked participants to define the new words; if unsuccessful, the child was given a contextual clue in the form of a sentence containing the word taken from the story, and then asked to define the word again. If the child was still unable to offer a suitable definition, they were given a forced-choice comprehension task, in which the correct definition was listed among four possible answers.

Assessing whether a word has been acquired is more complex than establishing whether its meaning is known, however. When a new item is entered into the lexicon, it is not only the word's semantics that are stored, but also its phonology, syntactic role, pragmatic uses and, as the child becomes literate, its orthography. Very few studies have probed whether children learn a word's syntactic properties when they hear the word in a story context. One exception was reported by Dickinson (1984), who explored 6- and 11-year-olds' ability to learn new words that they heard in conversations or stories or were explicitly taught. There were four components to the test phase: a “word recognition” task, a definition task, a comprehension test and a syntactic judgment task, the last of which required participants to distinguish correct uses of the newly-learned vocabulary from instances where the word was used as an inappropriate part of speech. The younger group's performance on the word recognition and comprehension tests demonstrated that they had acquired at least a partial representation of the meanings of the words they had encountered in stories. However, age differences were seen in children's ability to produce appropriate definitions and identify syntactically-appropriate usages of words, with only the older group succeeding on these tasks.

The current study builds on this previous work by exploring the nature of the representations young school-aged children form for new high-level vocabulary encountered while listening to stories. As in Wilkinson and Houston-Price (2013), the new vocabulary was introduced through repeated readings of a storybook by class teachers at school. Children listened to the same story three times, once a week for 3 weeks. The story contained nine new words, selected to be well beyond the vocabulary level expected for this age group and, to optimize learning, a definition was provided for each new word at its first mention in the story. In order to explore how easily words from different classes are acquired, the target vocabulary included nouns, verbs and adjectives. We assessed children's learning in three ways. As in Wilkinson and Houston-Price (2013), we conducted a forced-choice comprehension test using standardized vocabulary test picture cards, to establish whether the child had acquired at least a gist understanding of each word's meaning. Children were then asked to provide a definition of each target word, which we considered to provide a more stringent test of their understanding of the word's meaning. Finally, we included a grammaticality judgment task similar to that employed by Dickinson (1984), to assess the child's awareness of the new words' syntactic roles. The forced-choice comprehension task was always completed first; as the easiest of the three tasks, we anticipated that this would help children remain motivated to complete the remaining tasks. The grammaticality judgment task was always completed last, so that, if children were able to glean anything of the semantics of the words from their usage in this task, this would not affect their performance on the two comprehension tasks. We hypothesized that children would show greater knowledge of the target vocabulary than of a matched set of control words in all three tasks, demonstrating that they had incorporated both semantic and syntactic properties of the words into their lexicon. We expected that the older group would perform significantly better than the younger group on the definition production and syntactic awareness tasks.

## Methods

### Participants

One hundred and forty-eight children were recruited from 13 classes at three primary schools in the South of England. Two children declined to participate in all tests of their word knowledge and the data from a child with a genetic disorder were excluded from analyses. Remaining participants were 75 children in 6 Reception classes (mean age = 4 years, 9 ms; range = 4 years 3 ms—5 years 4 ms; 41 boys; henceforth “the 4-year-old group”) and 70 children in 7 Year 2 classes (mean age = 6 years 9 ms, range = 6 years 3 ms—7 years 4 ms; 32 boys; henceforth “the 6-year-old group”). The children's 13 class teachers, who were all native English speakers, also participated in the study. Teachers confirmed that listening to stories was a daily occurrence for the children in their classrooms.

### Materials

#### Target vocabulary

Three lists of words—Difficult Word List 1, Difficult Word List 2 and Easy words—were constructed from the test words used in the British Picture Vocabulary Scale 3rd Edition (BPVS-III; Dunn et al., 2009), so that each list comprised three nouns, three verbs, and three adjectives (see Appendix A). The words in the two difficult word lists were randomly selected (without replacement) from words of the relevant classes in sets 7–10 of the BPVS-III; these sets are suitable for testing the vocabulary knowledge of children aged from 9 to 14+ years and were expected to be largely unfamiliar to our participants. The assignment of the two lists as target and control words was counterbalanced so that approximately half of the children were trained on Difficult Word List 1 and half were trained on Difficult Word List 2; the list that was not selected to be trained provided the control words for each child. Easy words were selected from sets 1 and 2 of the BPVS-III (with the exception of one word taken from set 6, due to the lack of adjectives in the test's lower sets). BPVS-III sets 1 and 2 are suitable for testing the vocabulary knowledge of children aged between 2 and 4 years and were expected to be known by our participants. Easy words were included to motivate children to complete the tasks, and to enable us to check that children understood the requirements of each task.

#### Stories

A story was constructed to introduce the vocabulary in each of the difficult word lists. *Jade and Riley's Trip to the Zoo* included the words in Difficult Word List 1, while *Charlie's First Holiday* included the words in Difficult Word List 2. Stories were approximately matched in length and style (1227 and 1263 words respectively; for samples, see Appendix B). Target words were heard three times within the relevant story in a distributed and pseudo-random order, as required to create a coherent narrative. On the first occasion each word was heard, it was accompanied by a dictionary definition of the word taken from the Oxford Children's Dictionary (Allen, 2003) or Oxford Junior Dictionary (Dignen, 2003). Stories were printed on a piece of A3 card with a single picture on the back for children to look at while they were listening; the pictures did not illustrate the target vocabulary.

#### Test materials

Standard BPVS-III (Dunn et al., 2009) test cards were used to assess children's comprehension of the words in the three lists. Each test card displayed four pictures, one representing the target word. For the grammaticality judgment task, a syntactically-correct and syntactically-incorrect sentence was created for each word in the three lists. Sentences were short and simple and were unrelated in their content to the stories in which they had been heard. The syntactically-incorrect sentences were generated by presenting the target word as an inappropriate part of speech, following Dickinson (1984). For example, when a noun or adjective was used as a verb, this was indicated by the addition of the regular past tense ending. If a verb or adjective was used as a noun, it was pluralized, and when nouns or verbs were used as adjectives, they were inserted between the determiner and noun in a noun phrase construction (see Appendix C).

### Procedure

#### Listening phase

Each class was assigned to listen to one of the two stories, with the assignment counterbalanced within each school and year group; 3 Reception classes and 3 Year 2 classes heard the story containing the vocabulary in Difficult Word List 1, while 3 Reception and 4 Year 2 classes heard the story containing the vocabulary in Difficult Word List 2. Class teachers read the assigned story to their whole class during their usual story time once a week for 3 consecutive weeks. Children were able to see the picture on the back of the story card while it was read. Teachers were instructed to read the story in the same way they would usually read to their class during regular story time activities, but were asked to avoid providing any information about the vocabulary or content of the story other than that given in the script. A researcher was present at the first reading of each story to ensure that teachers understood and followed the instructions provided. At the end of the reading period, teachers confirmed that they had read the stories as instructed and had been able to avoid answering any questions children posed about the stories.

#### Test phase

Within 1 week of the final reading of the story, a researcher assessed children's knowledge of the new vocabulary. Testing was completed individually in a quiet area away from the classroom. Children completed the three tasks in a fixed order: the forced-choice comprehension task, followed by the definition production task and the grammaticality judgment task. Each task assessed children's knowledge of 27 words: the nine difficult “target” words contained in the story to which the child had been exposed; the nine difficult “control” words contained in the story to which the child had not been exposed; and the nine easy words, which the child was expected to know. Each child was assigned to one of four randomly-generated orderings of the 27 words, and all three tests were conducted in this order.

#### Forced-choice comprehension task

Children were presented with the relevant BPVS-III test card for each word and were asked to point to the picture that matched the corresponding test word. Responses were coded as either correct or incorrect.

#### Definition production task

Children were verbally presented with one test word at a time and were asked to say what they thought the word meant. If the child said they did not know, they were asked to guess and, if a partially correct response was given, the child was asked if they knew anything else about the word. Responses were recorded and later coded as either correct or incorrect by two independent researchers, on the basis of their similarity to the word's dictionary definition and the general sense of the child's response. Discrepancies between coders were discussed and agreement was reached in all cases.

#### Grammaticality judgment task

Children were introduced to two identical finger puppets who “sometimes say things in the right way, and sometimes say things in a silly way.” The researcher then produced the two sentences containing each target word as if one sentence was spoken by each puppet. One sentence in each pair was grammatically appropriate, the other grammatically inappropriate (see Appendix C). The child was asked to indicate which puppet was “right” and which was “silly” in each case. The puppet producing the grammatical sentence was randomly assigned on each trial. Children's responses to each trial were coded as either correct or incorrect.

On completion of each task, the child was offered a sticker of their choice as a reward and to provide a short break. The entire test session lasted around 20 min.

## Results

All 145 participants completed the forced-choice comprehension task. Two children failed to complete the definition production task (both 4-year-old girls) and nine children failed to complete the grammaticality judgment task (eight 4-year-olds, 2 girls and 6 boys; one 6-year-old girl). These children's data were included in analyses of the tasks they completed.

Preliminary analyses confirmed that very similar patterns of performance were shown by the children who listened to each of the two stories. We therefore collapsed the data across book group in our primary analyses of performance on each task, but we report any notable differences in the performance of the children who heard the two stories where these occurred.

### Forced-choice comprehension task

Table 1 presents the mean number of correct responses for each set of words in the comprehension task. For the Easy words, performance was at ceiling and significantly above the chance value of 2.25 (the value expected if children had chosen randomly from the four picture choices for each of the nine words tested) in both age groups [4-year-olds: *t*_(74)_ = 67.6, *p* < 0.001, Cohen's *d* = 15.72; 6-year-olds: *t*_(69)_ = 132.3, *p* < 0.001, Cohen's *d* = 31.85], demonstrating that children understood the requirements of the task.

**Table 1:** **Mean number of correct responses to each set of words in the forced-choice comprehension task**.

			**4- year-olds**		**6-year-olds**		**All children**	
			***N* = *75***		***N* = *70***		***N* = *145***	
		***N* words**	**Mean**	***SD***	**Mean**	***SD***	**Mean**	***SD***
Target words (Trained)	Nouns	3	2.00	0.81	2.64	0.57	2.31	0.77
	Verbs	3	1.53	0.95	2.16	1.00	1.83	1.02
	Adjectives	3	1.31	0.82	2.06	0.81	1.67	0.90
	All words	9	4.84	1.89	6.86	1.78	5.81	2.09
Control words (Untrained)	Nouns	3	1.20	0.82	2.17	0.15	1.67	1.01
	Verbs	3	0.80	0.82	1.64	1.01	1.21	1.01
	Adjectives	3	0.91	0.81	1.71	0.92	1.30	0.95
	All words	9	2.91	1.63	5.53	2.23	4.17	2.34
Easy words	Nouns	3	2.88	0.37	2.99	0.12	2.93	0.28
	Verbs	3	2.93	0.25	3.00	0.00	2.97	0.18
	Adjectives	3	2.63	0.49	2.80	0.40	2.71	0.46
	All words	9	8.44	0.79	8.79	0.41	8.61	0.66

To establish whether children in each age group had learned the meanings of the target vocabulary, a 2 (Age: 4-year-olds vs. 6-year-olds) × 2 (Training: Target vs. Control words) mixed ANOVA was conducted on children's total scores. There was a main effect of Age, *F*_(1, 143)_ = 73.2, *p* < 0.001, partial eta squared = 0.34; older children responded correctly to more words than younger children. There was also a main effect of Training, *F*_(1, 143)_ = 105.5, *p* < 0.001, partial eta squared = 0.43; children responded correctly to more target words than control words, showing that they had learned something about the meaning of the words while listening to the stories. The Age × Training interaction failed to reach significance, *F*_(1, 143)_ = 3.63, *p* = 0.059, partial eta squared = 0.03, showing that the two age groups benefited similarly from listening to the stories, according to this measure.

Word class (noun, verb, or adjective) was not included as a factor in the ANOVA. As the words in each class were not matched for their psycholinguistic properties (length, imageability, frequency, etc), we considered that it would be inappropriate to directly compare children's learning of the words in each class in order to draw conclusions about possible differences in children's ability to learn specific word types. However, to explore whether the learning children showed for words as a whole was also true of each individual word class, we compared children's performance on the target and control words of each word class separately. All comparisons were significant. Children in both age groups produced more correct responses to target words than control words, whether these were nouns [4-year-olds: *t*_(74)_ = 7.96, *p* < 0.001, partial eta squared = 0.46; 6-year-olds: *t*_(69)_ = 4.32, *p* < 0.001, partial eta squared = 0.21], verbs [4-year-olds: *t*_(74)_ = 5.02, *p* < 0.001, partial eta squared = 0.25; 6-year-olds: *t*_(69)_ = 3.58, *p* = 0.001, partial eta squared = 0.16] or adjectives [4-year-olds: *t*_(74)_ = 3.33, *p* = 0.001, partial eta squared = 0.13; 6-year-olds: *t*_(69)_ = 2.94, *p* = 0.004, partial eta squared = 0.11].

Finally, when we included Book Group as a factor in our overall analysis of variance, there was a weak interaction between Book Group, Age and Training, *F*_(1, 141)_ = 5.63, *p* = 0.019, partial eta squared = 0.04. *Post-hoc* analyses revealed that, while the learning shown by the younger group (calculated as the difference in their performance on target and control words) was equivalent for the two Book Groups, *F*_(1, 73)_ = 0.85, *p* = 0.36, partial eta squared = 0.01, the older group who heard the story containing Difficult Word List 2 showed greater learning than children who heard the story containing Difficult Word List 1, *F*_(1, 68)_ = 5.74, *p* = 0.019, partial eta squared = 0.08. However, importantly, children in both Book Groups showed large, significant differences in their scores for target and control words at both ages (all *p*s < 0.006), confirming that the general pattern of learning reported above was true, regardless of the specific story heard.

The results of the comprehension task therefore demonstrate that listening to stories containing difficult vocabulary enabled children to select the correct representations of the target words from a choice of four pictures. Learning was equivalent for the two age groups and evident for all three word types tested.

### Definition production task

Table 2 presents the mean number of appropriate definitions provided for each set of words. For Easy words, performance was again at ceiling for the older group and close to ceiling for the younger group; in each case, children produced appropriate definitions for around 8 of the 9 words in this set, showing that they understood the task's requirements.

**Table 2:** **Mean number of appropriate definitions provided for each set of words in the definition production task**.

			**4-year-olds**		**6-year-olds**		**All children**	
			***N* = *73***		***N* = *70***		***N* = *143***	
		**N words**	**Mean**	***SD***	**Mean**	***SD***	**Mean**	***SD***
Target words (Trained)	Nouns	3	1.34	0.97	2.24	0.91	1.78	1.04
	Verbs	3	0.97	1.01	1.89	1.07	1.42	1.13
	Adjectives	3	1.18	1.02	2.14	1.01	1.65	1.12
	All words	9	3.49	2.46	6.27	2.36	4.85	2.78
Control words (Untrained)	Nouns	3	0.64	0.77	1.57	0.94	1.10	0.97
	Verbs	3	0.29	0.59	1.29	1.04	0.78	0.97
	Adjectives	3	0.51	0.71	1.14	0.87	0.82	0.85
	All words	9	1.44	1.51	4.00	2.00	2.69	2.18
Easy words	Nouns	3	2.89	0.36	2.97	0.17	2.93	0.28
	Verbs	3	2.85	0.36	2.99	0.12	2.92	0.28
	Adjectives	3	2.03	0.78	2.54	0.65	2.28	0.76
	All words	9	7.77	1.06	8.50	0.70	8.13	0.97

To explore children's ability to produce appropriate definitions for the more difficult word sets, a 2 (Age: 4-year-olds vs. 6-year-olds) × 2 (Training: Target vs. Control words) mixed ANOVA was conducted on children's total scores for this task. Results mirrored those reported above for the forced-choice comprehension task. There was a main effect of Age, *F*_(1, 141)_ = 69.9, *p* < 0.001, partial eta squared = 0.33; older children produced more correct definitions than younger children. There was also a main effect of Training, *F*_(1, 141)_ = 202.5, *p* < 0.001, partial eta squared = 0.59; children produced more appropriate definitions for target words than control words. There was no Age × Training interaction, *F*_(1, 141)_ = 0.51, *p* = 0.48, partial eta squared = 0.00, demonstrating that the two age groups benefited similarly from listening to the stories in terms of their ability to produce appropriate definitions of the new words.

We again compared children's performance on the Target and Control words of each word class separately. All comparisons were significant. Children in both age groups produced more correct definitions of target words than control words, whether these were nouns [4-year-olds: *t*_(72)_ = 5.69, *p* < 0.001, partial eta squared = 0.31; 6-year-olds: *t*_(69)_ = 5.60, *p* < 0.001, partial eta squared = 0.31], verbs [4-year-olds: *t*_(72)_ = 5.95, *p* < 0.001, partial eta squared = 0.33; 6-year-olds: *t*_(69)_ = 4.15, *p* < 0.001, partial eta squared = 0.20) or adjectives [4-year-olds: *t*_(72)_ = 5.73, *p* < 0.001, partial eta squared = 0.31; 6-year-olds: *t*_(69)_ = 6.75, *p* < 0.001, partial eta squared = 0.40].

In this task, performance was affected by the book children had heard. When we included Book Group as a factor in our overall analysis of variance, there was a significant Book Group × Training interaction, *F*_(1, 139)_ = 11.4, *p* = 0.008, partial eta squared = 0.05. *Post-hoc* analyses revealed that children who heard the story containing Difficult Word List 1 showed a greater difference in their performance on target and control words than children who heard the story containing Difficult Word List 2, *F*_(1, 141)_ = 7.54, *p* = 0.007, partial eta squared = 0.05. However, and importantly, children in both Book Groups showed large, significant differences in their ability to define target and control words at both ages (all *p*s < 0.001), confirming that the general pattern of learning reported above was true regardless of the specific story children heard during the exposure phase.

Thus, listening to stories containing difficult vocabulary enabled children to produce appropriate definitions of the target words. Learning was again equivalent for the two age groups and evident for all three word classes

### Grammaticality judgment task

Table 3 presents the mean proportion of correct responses for each set of words on the grammaticality judgment task. Scores for this task were converted to proportions to acknowledge the differing numbers of target and control word test trials that remained after the test trial for one word was excluded (see Appendix C for further details). One sample *t*-tests confirmed that children in both age groups performed significantly better than the chance value of 0.5 (expected if children chose one of the two puppets randomly on each trial) on the Easy Word trials [4-year-olds: *t*_(66)_ = 3.77, *p* < 0.001, Cohen's *d* = 0.93; 6-year-olds: *t*_(68)_ = 16.0, *p* < 0.001, Cohen's *d* = 3.88), showing that participants in both age groups understood the requirements of the task.

**Table 3:** **Mean proportion of correct responses for each set of words in the grammaticality judgment task**.

		**4-year-olds**		**6-year-olds**		**All children**	
		***N* = *67***		***N* = *69***		***N* = *136***	
		**Mean**	***SD***	**Mean**	***SD***	**Mean**	***SD***
Target words (Trained)	Nouns	0.57	0.32	0.87	0.19	0.72	0.30
	Verbs	0.55	0.35	0.75	0.31	0.65	0.34
	Adjectives	0.54	0.34	0.83	0.23	0.68	0.33
	All words	0.56	0.20	0.81	0.17	0.69	0.23
Control words (Untrained)	Nouns	0.59	0.28	0.83	0.21	0.71	0.28
	Verbs	0.53	0.31	0.71	0.30	0.62	0.31
	Adjectives	0.51	0.39	0.76	0.25	0.64	0.30
	All words	0.54	0.18	0.77	0.16	0.66	0.20
Easy words	Nouns	0.56	0.27	0.84	0.23	0.70	0.29
	Verbs	0.60	0.29	0.84	0.22	0.72	0.28
	Adjectives	0.62	0.31	0.83	0.24	0.72	0.30
	All words	0.59	0.20	0.83	0.17	0.71	0.22

Scores on this task were converted to proportions to take account of the differing number of target and control word trials included for different participants (see Appendix C for more details).

To establish whether hearing words in stories supported children in discovering the words' grammatical roles, a 2 (Age: 4-year-olds vs. 6-year-olds) × 2 (Training: Target vs. Control words) mixed Anova was conducted on children's scores for this task. There was a main effect of Age, *F*_(1, 134)_ = 92.8, *p* < 0.001, partial eta squared = 0.41; older children produced more correct responses than younger children. There was no main effect of Training, *F*_(1, 134)_ = 2.66, *p* = 0.11, partial eta squared = 0.02, suggesting that children did not learn the new words' grammatical roles while they were listening to the stories. This general pattern was corroborated by comparisons of children's scores for the target and control words of each individual word class (all *p*s > 0.07). The ANOVA also found no Age × Training interaction, *F*_(1, 134)_ = 1.03, *p* = 0.31, partial eta squared = 0.01. However, to fully explore our hypothesis that older children would be better able to identify the syntactic category of the words they heard in stories, we conducted planned comparisons of each age group's performance on target and control words in this task. A weak Training effect was shown by the older group, who were better able to identify the correct grammatical usage of the target words than the control words, *t*_(68)_ = 2.30, *p* = 0.024, partial eta squared = 0.07. No effect of Training was shown by the younger group, *t*_(66)_ = 0.37, *p* = 0.71, partial eta squared = 0.002.

To explore whether the lack of a Training effect among the younger group reflected random responding on the difficult word trials of this task (perhaps due to fatigue, as this was the last task to be completed), one sample *t*-tests compared children's scores to chance (0.5). In both age groups, performance was above chance for both target words [4-year-olds: *t*_(66)_ = 2.24, *p* = 0.029, Cohen's *d* = 0.55; 6-year-olds: *t*_(68)_ = 15.5, *p* < 0.001, Cohen's *d* = 3.76] and control words [4-year-olds: *t*_(66)_ = 2.00, *p* = 0.05, Cohen's *d* = 0.49; 6-year-olds: *t*_(68)_ = 14.1, *p* < 0.001, Cohen's *d* = 3.42]. These results show that children were not responding randomly on this task; at least some of the children in each age group were able to indicate how the difficult vocabulary should be used in sentences. Rather, the lack of a Training effect among the younger group indicates that hearing the target vocabulary in stories did not facilitate this group's performance on this task.

In sum, while children overall showed no learning of the grammatical role of the target vocabulary in this study, the older group showed greater awareness of the appropriate usage of the vocabulary to which they had been exposed than of the matched control words. There were no effects of the specific story to which children had been exposed in this task, and no evidence of learning for any individual word type.

### Between- and within-task relationships

To establish the extent to which the three tasks assessed the same or different components of children's learning about the target vocabulary, we first examined the relationships between children's performance on each task. For each task, the “number of words learned” was computed as the difference between the child's scores for target and control words. As we explored the between-task relationships both for all words combined and for individual word classes, and for both children overall and for the two age groups separately, alpha was set at.0125 (.05/4) for these analyses.

When all children and all word types were included in the analysis, there was a significant correlation between the number of words learned in the forced-choice and definition production tasks, *r*_(143)_ = 0.31, *p* < 0.001, suggesting that these were tapping into the same aspects of children's knowledge of the target vocabulary. There was no relationship between the learning shown on the forced-choice and grammaticality judgment tasks, *r*_(136)_ = 0.11, *p* = 0.22. However, performance on the definition production and grammaticality judgment tasks was correlated, *r*_(135)_ = 0.25, *p* = 0.003, showing that those children who had learned the grammatical use of the target vocabulary were also better able to define these words.

We explored these relationships further by computing the measure of learning for each word class separately. Interestingly, the relationship reported above between the two comprehension tasks was found to be specific to word class. That is, we found correlations between the learning children showed on the forced-choice and definition production tasks for nouns, *r*_(143)_ = 0.34, *p* < 0.001, verbs, *r*_(143)_ = 0.43, *p* < 0.001, and adjectives, *r*_(143)_ = 0.25, *p* = 0.002, and no cross-word class relationships between tasks (all *p*s > 0.08). Thus, the relationships we observed do not simply reflect the more able children doing better in both tasks. Rather, the picture-selection task and definition production task appear to have tapped into the same aspects of children's knowledge of the target vocabulary, presumably semantic awareness. The correlations between the learning shown on the definition production task and grammaticality judgment task did not meet our adjusted alpha criterion for any individual word class [nouns: *r*_(135)_ = 0.16, *p* = 0.057; verbs: *r*_(135)_ = 0.18, *p* = 0.038; adjectives: *r*_(135)_ = 0.13, *p* = 0.13], but again there were no cross-word class relationships (all *p*s > 0.2).

When the learning shown by the two age groups was explored separately, some interesting differences were seen in the pattern of between-task relationships. The younger group showed a very strong relationship between their learning on the forced-choice and definition production tasks, *r*_(73)_ = 0.51, *p* < 0.001. As for children overall, this relationship was underpinned by strong word class-specific relationships [nouns: *r*_(73)_ = 0.44, *p* < 0.001; verbs: *r*_(73)_ = 0.34, *p* = 0.004; adjectives: *r*_(73)_ = 0.43, *p* < 0.001). The younger group revealed no relationships between their performance on the definition production and grammaticality judgment task, either overall or for any individual word class (all *p*s > 0.06). In contrast, the older group showed no relationship between their overall scores for the two comprehension tasks, *r*_(70)_ = 0.11, *p* = 0.36, and only a weak relationship between their scores for the definition production and grammaticality judgment tasks, which did not meet our criterion for alpha, *r*_(69)_ = 0.28, *p* = 0.02. However, when words were separated by word class, relationships were found between the older group's learning of verbs in the forced-choice and definition production tasks, *r*_(70)_ = 0.51, *p* < 0.001, and in the definition production and grammaticality judgment tasks, *r*_(69)_ = 0.33, *p* = 0.006. No significant cross-word class relationships were shown by either age group.

Given the word-class specificity of the between-task relationships reported above—whereby performance on the words of any word class in one task was only ever correlated with performance on the same word class in another task—we went on to examine whether there were any within-task relationships between performance on the three word categories included in our study. As we examined the within-task correlations both for children overall and for each age group separately we set our alpha criterion at 0.025 (0.05/2) for these analyses. There were no positive cross-word class correlations in performance on any of the three tasks, either for children overall or for either individual age group (all *p*s > 0.05). Indeed, the only correlation that met our criterion for alpha was a negative relationship between children's performance on verbs and adjectives in the grammaticality judgment task, *r*_(136)_ = −0.21, *p* = 0.02.

In sum, performance on the two comprehension tasks was strongly related, especially among the younger participants. In contrast, performance on the picture-selection task and grammaticality judgment task was unrelated, suggesting that these assess entirely independent components of word knowledge, likely to be semantic and syntactic knowledge respectively. The ability to produce definitions of newly-learned words was related to grammatical awareness in children as a whole, but the relationship was most evident in the older group's ability to define and recognize the appropriate usage of verbs. The systematic lack of cross- word class relationships demonstrates that children's ability to learn one word class while listening to stories was, surprisingly, unrelated to their ability to learn other word classes.

## Discussion

This study assessed the learning children showed for the vocabulary they had encountered while listening to stories using three tests of word knowledge. In a forced-choice comprehension task, children were asked to select the picture that represented each word from a set of four candidate pictures. As hypothesized, children in both age groups (4- and 6-year-olds) gave the correct response significantly more often for exposed words than for a matched set of control words, with no difference between the age groups. Recall that children were not provided with pictures to support their story comprehension during the listening phase; their success at the forced-choice task therefore indicates that hearing the words (and their accompanying definitions) in stories supported the construction of lexical representations that were sufficient to identify the relevant pictorial representation for each word (or, possibly, to rule out the pictures that were unrelated to the words).

The same pattern was seen in the definition production task, which required children to explain the meanings of the newly-learned vocabulary; more appropriate definitions were given for target words than control words. Hearing new words (and their accompanying definitions) in stories therefore enabled the children to make a reasonable attempt at defining their meanings, suggesting that the lexical representations they had established for these words comprised more than just a gist interpretation. It is worth noting that, in contrast to the forced-choice task, performance on the definition task would have been directly supported by the manner of presentation of the target vocabulary in the stories; a child could have succeeded at this task by simply recalling the definitions that had been provided within the story. We would consider such an ability to constitute a form of word learning. When children were listening to the stories, they were not expecting to be tested on the meaning of the new vocabulary these contained; that they remembered the definitions included in the stories, and could produce these as appropriate when they were later asked to define each word, in our view, provides a clear indication that learning had taken place. Contrary to our expectations, the two age groups performed equally well on this more challenging comprehension task (c.f. Dickinson, 1984). Thus, from the earliest school years, children are able to ascertain sufficient detail of the meanings of words they encounter in stories to be able to describe what these mean, even for vocabulary items considerably beyond the level expected for their age group. These findings corroborate previous evidence that children are able to acquire the meanings of difficult vocabulary items when listening to stories that provide semantic support for words' meanings (Elley, 1989; Sénéchal et al., 1995; Brett et al., 1996; Sénéchal, 1997; Reese and Cox, 1999; Penno et al., 2002; Justice et al., 2005; Biemiller and Boote, 2006; Beck and McKeown, 2007; Wilkinson and Houston-Price, 2013), and extend this evidence to a younger group of 4-year-olds who have only recently started school.

We also assessed children's ability to recognize the appropriate grammatical usage of the exposed vocabulary through a forced-choice puppet task. Each word was placed into two sentence frames containing no semantic clues to the word's meaning, where one frame was syntactically appropriate, the other syntactically inappropriate; children were asked to indicate which puppet said it “right” and which was “silly.” As a whole, children identified the correct uses of the target words no better than the correct uses of the control words, with performance on both word lists superior to chance. One possible explanation of these findings is that children had heard the words in the difficult word lists before, even if they did not know what they meant. If grammatical class is the first piece of information children acquire about a word when it is heard in a sentence context, they might have already been aware of the syntactic roles of the difficult vocabulary we set out to teach them. However, performance was not at ceiling on this task, leaving plenty of room for learning to result from the additional exposure children gained when they heard the words in the stories. *Post-hoc* analyses suggested that the older group did, in fact, perform better on target word trials than control word trials, suggesting that at least some 6-year-old children had incorporated some information about the exposed words' syntactic roles into their lexical representations for these items (c.f. Dickinson, 1984).

Additional analyses explored whether the three tasks that formed our assessment battery indexed the same or different components of children's knowledge of the target vocabulary. There was no shared variance between children's performance on the forced-choice comprehension task and grammaticality judgment task, confirming that the components of word knowledge addressed by these tasks were entirely independent. Moreover, the lack of relationship between these tasks means that one cannot attribute the positive correlations we observed—between the two comprehension tasks and between the definition production and grammaticality judgment tasks—simply to “brighter” children doing better across the board. Rather, these positive relationships suggest that the picture-selection task and definition task both tapped children's awareness of the semantic properties of the newly-learned vocabulary, while the definition and grammaticality judgment tasks both provided an index of the child's knowledge of the words' syntactic roles. We had included the definition task in our assessment battery as a more challenging test of semantic knowledge than was offered by the picture-selection task, not anticipating that this task might also reflect the child's awareness of the word's syntactic role. However, it makes sense that, in order to produce a reasonable account of what a word means, a child would need to know something about how the word might be used—in other words, its grammatical role. Furthermore, returning to the issue of how the stories might have supported children's performance on the definition task, the relationships found between performance on this task and performance on the other tasks suggest that children were doing more than reeling off rote-learned definitions when asked to explain the target words' meanings. Rather, they were drawing on the same store of semantic and syntactic knowledge that supported their performance on the forced-choice and grammaticality judgment tasks respectively.

The design of our study also allowed us to explore whether the learning of difficult vocabulary in a range of word classes (nouns, verbs, and adjectives) is supported by introducing children to these words in stories. Previous research has reported differential rates of learning of different word classes when children listen to stories or instructional videos, such that nouns are acquired more easily than other word types (Robbins and Ehri, 1994; Ard and Beverly, 2004; Dockrell et al., 2007). For example, Dockrell et al. (2007) observed a “noun bias” in 4- to 7-year-old children's ability to produce scientific terms after children had been introduced to nouns, verbs and adjectives in an educational video. A similar pattern was observed in Dockrell et al.'s measures of comprehension; while children showed equivalent levels of understanding of the nouns and adjectives they had encountered, performance on these word classes was superior to their understanding of the verbs. A “noun bias” is also seen in early language development, when nouns dominate over verbs, adjectives and other word classes in infants' vocabularies (Goldin-Meadow et al., 1976; Waxman and Kosowski, 1990). Alternative explanations exist for this phenomenon; while some highlight the conceptual difficulty of representing properties of objects or relationships between objects compared to representing objects themselves (Gentner, 1978; Spelke, 1994), others point to the relative complexity of the lexical representations we hold for nouns, verbs and adjectives (Gillette et al., 1999).

The current study was unable to directly compare children's learning of words in different word classes, as we did not attempt to match the words of each type for properties that might have impacted on the ease with which they were learned. However, the results were clear in establishing that the sharing of stories can be used to introduce the full range of word types into children's lexicons. Children in both age groups showed a greater awareness of the meanings of exposed words in all three word classes in both the picture-selection and definition production tasks. Whether it is as easy to acquire grammatical knowledge about the three word types is less clear. While hearing the target vocabulary in the story context appeared to provide some support to 6-year-olds in judging whether the words were used in appropriate syntactic frames, their learning was not robust enough to be shown for any individual word class.

A particularly surprising finding in relation to children's learning of the words in different word classes was the lack of relationship between them. While we found systematic patterns of between-task correlations within each word class (such that picture-selection performance for nouns was strongly correlated with definition production performance for nouns, e.g.), there were no within- or between-task relationships that crossed word class, for children overall or either age group. Children's ability to learn the meanings of words in one syntactic category was, in this study, entirely unrelated to their ability to learn words in another. This finding appears to contradict the view that some children are simply better at acquiring new vocabulary (e.g., Daneman, 1988; Biemiller and Slonim, 2001). Rather, the results of the current study suggest that the learning of words in different classes may be supported by a distinct set of mechanisms or strategies.

Closer examination of the within-class correlations between the three assessments in our test battery provides some insight into the mechanisms that might support the representation of different word types. While the younger group showed within-class relationships between their performance on the two comprehension tasks for all three word types, the older children only showed such a relationship in assessments of their understanding of verbs. Similarly, the older group showed a relationship between their performance on the definition production and grammaticality judgment tasks only when they were assessed on their knowledge of verbs. If this pattern of relationships is interpreted in terms of the knowledge children were able to call upon when asked to produce a definition of each word, as discussed above, then it would appear that the younger children drew on specific semantic knowledge of the words in each class, as indexed by their scores for these items in the picture-selection task. In contrast, and only when they were asked to define verbs, the older group appear to have drawn on both their semantic knowledge of these words and their awareness of the words' syntactic roles, as indexed by children's scores on the grammaticality judgment task. This account suggests that syntactic information plays a central role in supporting children's understanding of verbs. Such an interpretation is congruent with the view that verbs are “special” because they define the structure of the sentences in which they appear (Levin, 1993), and that syntactic information is fundamental to the representation of this word class in the lexicon (Garrett, 1990). Indeed, some accounts claim that, when a verb is retrieved for production purposes, information about the sentence frames in which it might be used is automatically retrieved at the same time (Pickering and Branigan, 1998). Thus, it is feasible that the older group, for whom some evidence of learning was seen on the grammaticality judgment task, automatically retrieved information about the words' syntactic roles when asked to provide definitions of verbs, but not when they were asked to define nouns or adjectives. Further work is clearly needed to explore these ideas.

In addition to furthering our understanding of the components of the knowledge children acquire while listening to stories, and how these may differ for the vocabulary in different word classes, this work has clear implications for the way in which vocabulary instruction is delivered in educational contexts. A previous meta-analysis of research in this field found that teacher-delivered interventions tend to produce smaller effects sizes than experimenter-led studies of vocabulary learning (Mol et al., 2009) and the authors note the importance of addressing this difference in order to “bridge the gap between research and practice” (p. 1000). The results of the current study confirm that teacher-delivered stories to whole classes of children can effectively introduce difficult new vocabulary of a range of word types. Given teachers' established willingness to engage in shared story sessions as part of the literacy curriculum, an extension of this practice is likely to be both affordable and practicable for schools to deliver. To this end, we encourage the development of challenging but engaging reading materials that incorporate the high-level vocabulary that children may be missing. It is likely that, in addition to supporting the language development of children whose home environments might be considered linguistically impoverished, this practice would benefit the growing numbers of children in our schools for whom the language of instruction is not their first language. In a study with young primary school children, Biemiller and Boote (2006) found that both monolingual children and children learning English as an additional language (EAL) benefited from repeated storybook readings accompanied by explanations of the target vocabulary. Collins (2010) recently reported that the same factors support the acquisition of sophisticated vocabulary knowledge in EAL preschoolers.

Coyne et al. (2012) recently proposed an intensive 18-week programme of vocabulary instruction that teachers might deliver in two half-hour slots per week. The intervention aims to support children's learning of 54 “Tier 2” vocabulary items (Beck and McKeown, 2007) that are unlikely to be learned without support, and is based on the principles of repeated exposure to the target vocabulary through shared stories and explicit definition of words as they are encountered. Coyne et al. advise practitioners not to shy away from providing explicit vocabulary instruction due to concerns that any gains achieved will be insignificant in light of the large number of words children need to learn (Anderson and Nagy, 1992). Just as a snowball can cause an avalanche, the more words a child knows, the easier they will find it to learn further words (Stanovich, 1986; Biemiller and Slonim, 2001). While a child may not need “funambulist” in their lexicon unless they are planning to join the circus, the benefits of extending a child's word knowledge to a level beyond that needed for everyday conversation are indisputable (Cunningham and Stanovich, 1997; Storch and Whitehurst, 2002; Chall and Jacobs, 2003; Dickinson et al., 2003).

### Conflict of interest statement

The authors declare that the research was conducted in the absence of any commercial or financial relationships that could be construed as a potential conflict of interest.

## Appendix A

### Word lists and the bpvs-iii word set from which words were drawn (in brackets)

**Table:**

**Word Class**	**Difficult word list 1**	**Difficult word list 2**	**Easy words**
Noun	Aquarium (9)	Luggage (9)	Ball (1)
Noun	Bouquet (10)	Antlers (8)	Duck (1)
Noun	Hyena (7)	Valley (8)	Mouth (1)
Verb	Snarling (9)	Applauding (9)	Swimming (1)
Verb	Departing (10)	Harvesting (8)	Drinking (1)
Verb	Grooming (7)	Greeting (8)	Jumping (1)
Adjective	Exhausted (9)	Inflated (9)	Rough (6)
Adjective	Canine (10)	Adjustable (8)	Happy (2)
Adjective	Tubular (7)	Tropical (8)	Empty (2)

## Appendix B

### Samples from the stories used to introduce the target vocabulary

Sample from *Jade and Riley's Trip to the Zoo* (Difficult word list 1)

Jade was excited about seeing the monkeys and ran to their cage. Two monkeys were picking at each other's fur. Dad told Jade they were **grooming** each other. **Grooming means to clean and brush**. Jade thought the monkeys seemed to enjoy this. Riley liked the monkeys because they were having fun and none of them were **snarling** at him. Mum and Dad were feeling **exhausted**. **Exhausted means very tired**. So they decided it was a good time to have their picnic, and they sat down for their lunch. It was a special day out so Mum brought Riley and Jade some sweeties for after lunch, as a treat. These were their favourite, little circles of chocolate in a **tubular** shaped packet just like the tunnel they had walked through earlier… but much smaller! “Smarties!” shouted Jade. “My favourite” said Riley.

Sample from *Charlie's First Holiday* (Difficult word list 2)

Then they had to wait for ages in the waiting room. Charlie began to think about what Spain was like. “Is Spain a **tropical** country?” he asked. **A tropical place has a very hot, wet climate**. “No Charlie, Spain is hot but it doesn't rain much,” said Mum. While they were waiting for their flight, a man came and sat next to them wearing a funny hat with **antlers** on it. **Antlers are the branched horns of a deer**. At last, there was an announcement calling them to get on the aeroplane. Charlie was so excited. Two air stewardesses were **greeting** all the passengers and checking tickets as they got on the plane. One of them told Charlie that he couldn't keep his **inflated** crocodile blown up otherwise Croccy would need his own seat, and then they would have to buy him another ticket! So Charlie and his brother quickly squashed him flat and Mum put him in her bag. “See you on the other side, Croccy,” whispered Charlie.

## Appendix C

### Sentences used in the grammaticality judgment task

**Table:**

	**Grammatically correct**	**Grammatically incorrect**
Difficult word list 1	The **hyena** went hunting to feed his family	The **hyena** man went hunting to feed his family
	Mum and Dad went to the **aquarium**	Mum and Dad **aquariumed**
	Frankie bought his girlfriend a **bouquet**	Frankie **bouqueted** his girlfriend
	The cat sat by the fire **grooming** her kittens	The **grooming** fire warmed the cat
	On holiday in the jungle Jack saw a lion **snarling**	Jack saw **snarlings** in the jungle
	Whilst **departing** from the park the family laughed	At the park the family saw the **departings** laughing
	Mum had lots of **tubular** shaped cushions on her bed	Mum had lots of **tubulars** and cushions on her bed
	After a long day at work Dad was **exhausted**	The **exhausteds** had had a long day at work
	The **canine** animals walked through the forest	The animals **canined** through the forest
Difficult word list 2	Old man Jim lived in the **valley**	The **valley** door of Jim’s house was red
	Father Christmas’ reindeers have **antlers**	Father Christmas’ reindeers **antlered** through the sky
	Sophie took lots of **luggage** to Canada with her	The **luggage** plane flew Sophie to Canada
	Granny gave Karen a warm **greeting** when she got to her house^*^	The **greeting** house was where Granny lived
	The farmers were **harvesting** in the bright warm sunshine	The **harvesting** vegetables were collected by the farmer
	As the play finished the crowd were **applauding**	As the play finished the **applaudings** cheered
	Dad changed the **adjustable** volume on the radio	Dad **adjustabled** the volume on the radio
	Mrs Magoo lives in a **tropical** country	Mrs Magoo **tropicalled** to a different country
	At the birthday party there were lots of **inflated** balloons	At the birthday party there were lots of **inflateds**
Easy words	Timmy and Ben played catch with their shiny new **ball**	Timmy and Ben **balled** in the park
	Gemma fed bread to her pet **duck**	The **duck** pet asked Gemma for food
	The sleepy hippo yawned with his big **mouth**	The **mouth** hippo felt sleepy
	Harry liked **jumping** on his trampoline	The **jumpings** liked the trampoline
	Caitlin loved **drinking** milkshakes	The **drinkings** loved making milkshakes
	Barry likes to go **swimming**	The **swimming** children splashed in the pool
	Roya drank all of her milk and now her glass was **empty**	The **empties** drank all of their milk
	The **happy** elephant twizzled his trunk	The elephant **happied** back to his herd
	The **rough** ground in the garden hurt Grandad’s feet	Grandad **roughed** in the garden

* “Greeting” was erroneously used as a noun in the “grammatically correct” sentence for this word, when it had been used as a verb in the story. The test trial for this word was therefore excluded from analyses of children’s learning on this task. Note that “greeting” was a target word for some children and a control word for others (depending on the story they heard).
